# Development of Pharmacophore Model for Indeno[1,2-*b*]indoles as Human Protein Kinase CK2 Inhibitors and Database Mining

**DOI:** 10.3390/ph10010008

**Published:** 2017-01-09

**Authors:** Samer Haidar, Zouhair Bouaziz, Christelle Marminon, Tuomo Laitinen, Antti Poso, Marc Le Borgne, Joachim Jose

**Affiliations:** 1 Institut für Pharmazeutische und Medizinische Chemie, PharmaCampus, Westfälische Wilhelms-Universität Münster, Corrensstr. 48, 48149 Münster, Germany; joachim.jose@uni-muenster.de; 2 Faculty of Pharmacy, Damascus University, Damascus, P.O. Box 9411, Syria; 3 Université de Lyon, Université Claude Bernard Lyon 1, Faculté de Pharmacie—ISPB, EA 4446 Bioactive Molecules and Medicinal Chemistry, SFR Santé Lyon-Est CNRS UMS3453—INSERM US7, 8 Avenue Rockefeller, F-69373 Lyon CEDEX 8, France; zouhair.bouaziz@univ-lyon1.fr (Z.B.); christelle.marminon-davoust@univ-lyon1.fr (C.M.); marc.le-borgne@univ-lyon1.fr (M.L.B.); 4 School of Pharmacy, Faculty of Health Sciences, University of Eastern, 70211 Kuopio, Finland; tuomo.laitinen@uef.fi (T.L.); antti.poso@uef.fi (A.P.); 5 University Hospital Tübingen, Department of Internal Medicine VIII, Otfried-Müller-Straβe 10, 72076 Tübingen, Germany

**Keywords:** CK2, cancer, indeno[1,2-*b*]indoles, pharmacophore, MOE, ZINC database

## Abstract

Protein kinase CK2, initially designated as casein kinase 2, is an ubiquitously expressed serine/threonine kinase. This enzyme, implicated in many cellular processes, is highly expressed and active in many tumor cells. A large number of compounds has been developed as inhibitors comprising different backbones. Beside others, structures with an indeno[1,2-*b*]indole scaffold turned out to be potent new leads. With the aim of developing new inhibitors of human protein kinase CK2, we report here on the generation of common feature pharmacophore model to further explain the binding requirements for human CK2 inhibitors. Nine common chemical features of indeno[1,2-*b*]indole-type CK2 inhibitors were determined using MOE software (Chemical Computing Group, Montreal, Canada). This pharmacophore model was used for database mining with the aim to identify novel scaffolds for developing new potent and selective CK2 inhibitors. Using this strategy several structures were selected by searching inside the ZINC compound database. One of the selected compounds was bikaverin (6,11-dihydroxy-3,8-dimethoxy-1-methylbenzo[*b*]xanthene-7,10,12-trione), a natural compound which is produced by several kinds of fungi. This compound was tested on human recombinant CK2 and turned out to be an active inhibitor with an IC_50_ value of 1.24 µM.

## 1. Introduction

Cancer remains the leading cause of death in the world and around eight million deaths related to cancer were reported worldwide in 2012. The cancer cases are expected to increase sharply within the coming twenty years [1]. Different strategies for the treatment of cancer, including kinase inhibition [2] were employed and investigated to cure the disease or prolong life and increase its quality [3]. The first known protein kinase inhibitor was developed in the early 80s, and since then a large number of compounds has been described (for reviews see [3,4]). The first protein kinase inhibitor approved as drug was imatinib (Gleevec^®^, Novartis, Basel, Switzerland), which is used in the treatment of myelogenous leukemia [5]. To date, around one third of all drug discovery projects in pharmaceutical industry are related to protein kinase inhibitors [6]. Casein Kinase 2 (CK2) is a heterotetrameric enzyme which is ubiquitously expressed in mammalian cells [7]. CK2 enhances cancer phenotype by suppressing apoptosis and stimulating cell growth. Thus, inhibition of this enzyme can induce the physiological process of apoptosis, leading to tumor cell death [2]. Different backbones were used as skeleton for ATP competitive CK2 inhibitors, among them indeno[1,2-*b*]indoles have been identified as potent leads [8]. Several studies were published recently describing a large number of indeno[1,2-*b*]indoles and their activity as CK2 inhibitors [9,10,11,12,13]. Some of those compounds turned to be highly active with IC_50_ values in the low nanomolar range.

Molecular modeling is widely used for drug design and development, with the aim of finding new lead structures for the treatment of different diseases. Virtual screening or “mining” of databases is considered as an important tool in drug discovery. The aim in using in silico techniques is to reduce the costs and time to discover new active compounds, since reducing the period of a new drug from research laboratory to the market by one year can reduce the costs by millions of dollars. To develop a pharmacophore model is an important concept in computer-aided drug design (CADD), as this method can reduce the complexity of molecular interactions between compound and target to a set of few features. This method was successfully applied in drug discovery [14,15,16,17,18,19] and many molecular modeling software are available among them the Molecular Operating Environment (MOE). In this study a pharmacophore model for human protein kinase CK2 was constructed on the basis of 50 indeno[1,2-*b*]indoles and used to identify new hits by mining the ZINC compound database [20].

## 2. Results

In this work we used the inhibition data obtained with a set of 50 different indeno[1,2-*b*]indoles and human protein kinase CK2 [9,10,12,13]. We used these indeno[1,2-*b*]indoles, because they have all been tested on inhibition of human protein kinase CK2 in our lab with the same assay under identical conditions. We decided not to take any published inhibition data from other groups, because they were determined under different conditions (e.g., different assay, different ATP-concentration, substrate concentration, temperature etc.), and hence could lead to a blurred or less clear picture. The structures of the 50 compounds under investigation and their IC_50_ values are presented in Table 1, Table 2 and Table 3.

MOE software (Chemical Computing Group, Montreal, Canada) package was used to perform this study [21]. MOE has been proved to be an effective tool for pharmacophore construction [22], other alternative software in this field might be Catalyst, LigandScout, and Phase, which are also effective for pharmacophore modeling.The resulting test set contained 524 conformations of these 50 compounds. Inhibitors with IC_50_ values of less than 10 µM were considered as moderate or highly active compounds. All others were designated as none active. This filter resulted in a number of 26 active and 24 non active compounds.

**Figure 1:**
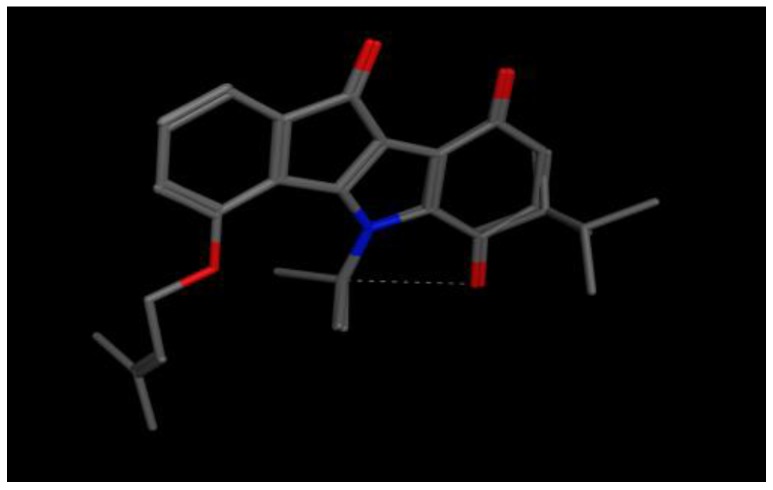
Alignment of compounds **1**–**5** from the training setof indeno [1,2-*b*]indole inhibitors of CK2, carbon atoms are in gray, oxygen atoms are in red, nitrogen atom in blue and intermolecular H-bond in dots.

The pharmacophore model was developed by aligning the structures of the five most active compounds (training set), which were very well aligned as it is clear in Figure 1 were the backbones of the compounds fits very well above each other due to structure similarity.

**Figure 2:**
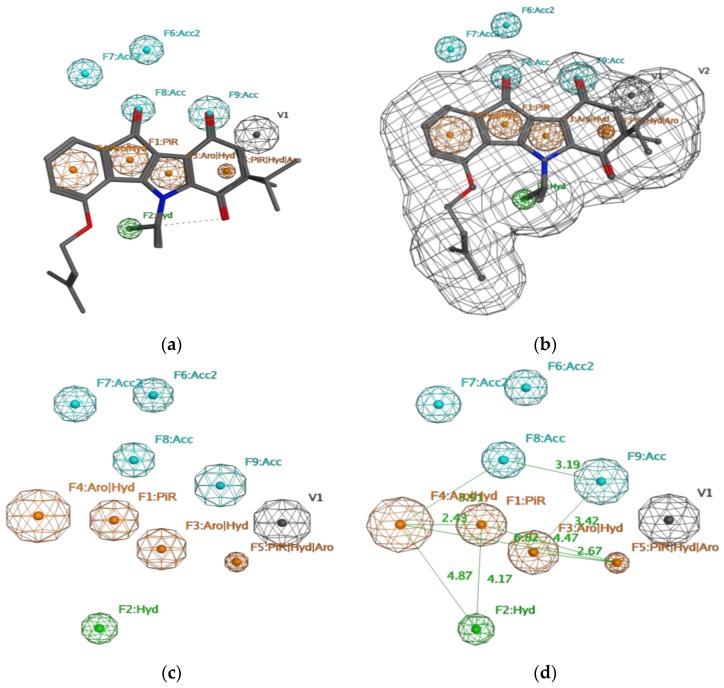
The developed pharmacophore model. (**a**) Alignment of common feature pharmacophore model with training set; (**b**) Alignment of common feature pharmacophore model with training set with occupied volume; (**c**) Common feature pharmacophore model of indeno[1,2-*b*]indole-type CK2 inhibitors; (**d**) Common feature pharmacophore model with the distance constraints. **Acc**: H-bond acceptor, **Acc 2**: H-bond acceptor projection, **Aro**: Aromatic center, **Hyd**: Hydrophobic centroid, **PiR**: π ring center.

The pharmacophore model derived thereof is shown in Figure 2. It is described by nine necessary features. In particular, a π ring center on the B ring, a hydrophobic centroid, two aromatic center/hydrophobic atoms on ring A and C, a π ring center/aromatic/hydrophobic centroid on ring D, and two hydrogen bond acceptors on rings B and D, together with two hydrogen acceptor projections, beside one excluded volume (no matching atom can be inside). Validity of the pharmacophore model was analyzed by its ability to identify the medium and highly active compounds only, which are represented by compounds **1**–**26**. It turned out that with this pharmacophore model it was possible to identify **22** out of the **26** designated as active compounds, which represents 85% of all. Compounds **9**, **13**, **17**, and **20** were not identified. Only four compounds out of the **24** non active compounds were selected and hence identified as false positive, which represents 17% of the non-active compounds. The compounds that were selected as false positives were compounds **29**, **31**, **36**, and **40**.

The nine features of this model as described were used to search in part of the ZINC database “All Now” (all purchasable in 2 weeks), with the aim of identifying an active CK2 inhibitor. However, by applying all the nine features of the developed pharmacophore model, it was not possible to identify any compound from the selected database. For that reason, the search was repeated using only eight out of nine features together with the excluding volume. The best result obtained was by omitting F2 (this feature is outside the main backbone of the indeno[1,2-*b*]indoles) and keeping all other features, which resulted in the selection of 57 compounds. The 57 compounds selected belong to different chemical backbones such as naphtho[2,3-*b*]furanedione, quino[2,3-*b*] acridinetetrone, naphthacenedione, and pyrazoloquinazoline. From this set of compounds, only two pyrrolizine based compounds were excluded from further observations, due to their inappropriate structure for enzyme inhibitors. They are rather fragment-like compounds according to “the role of three” filters (MW ≤ 300, cLogP ≤ 3, number of H-bond donors ≤ 3, number of H-bond acceptors ≤ 3) [23], which can be misleading for the development of active and selective inhibitors. The structures of the 55 selected compounds are shown in Supplementary Table S1. Surprisingly none of the selected compounds belonged to the indeno[1,2-*b*]indole group of compounds. As a control, the ZINC database was searched on the presence of indeno[1,2-*b*]indole like compounds and—to our surprise—it did not contain any.

**Figure 3:**
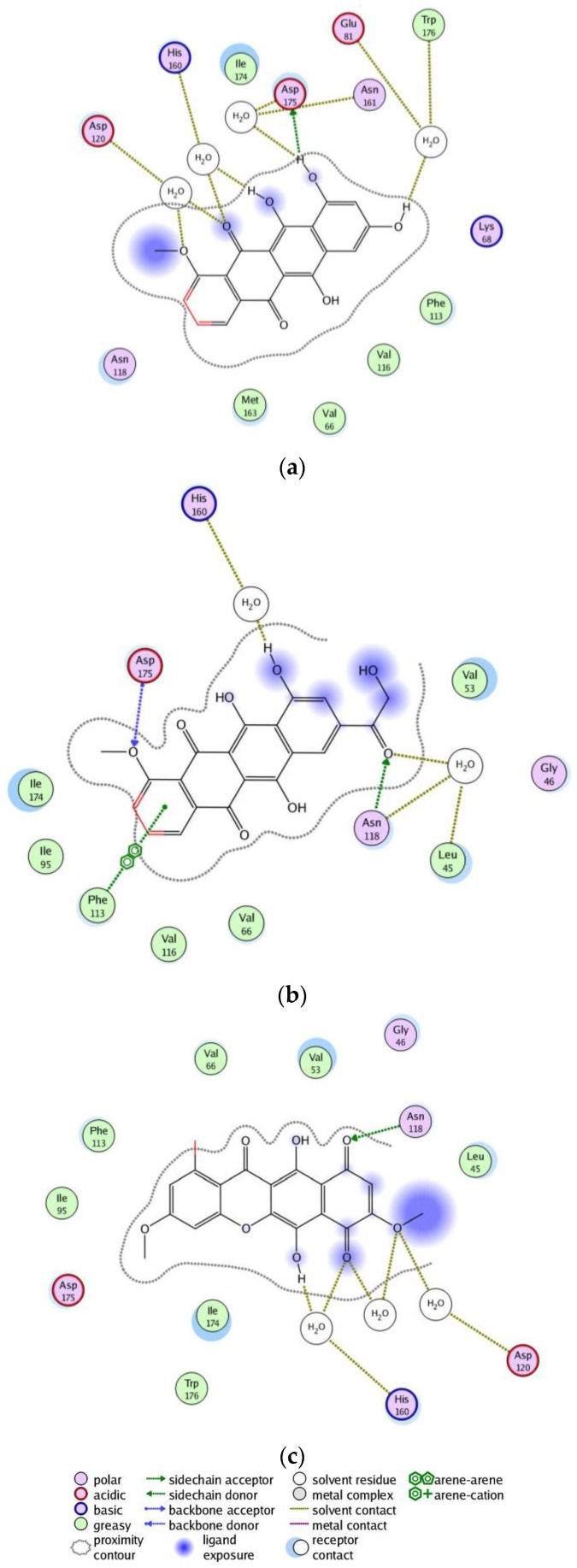
2-D interactions between the three best selected compounds and the ATP binding site. (**a**) ZINC35643753; (**b**) ZINC44136135; (**c**) ZINC05765165.

**Figure 4:**
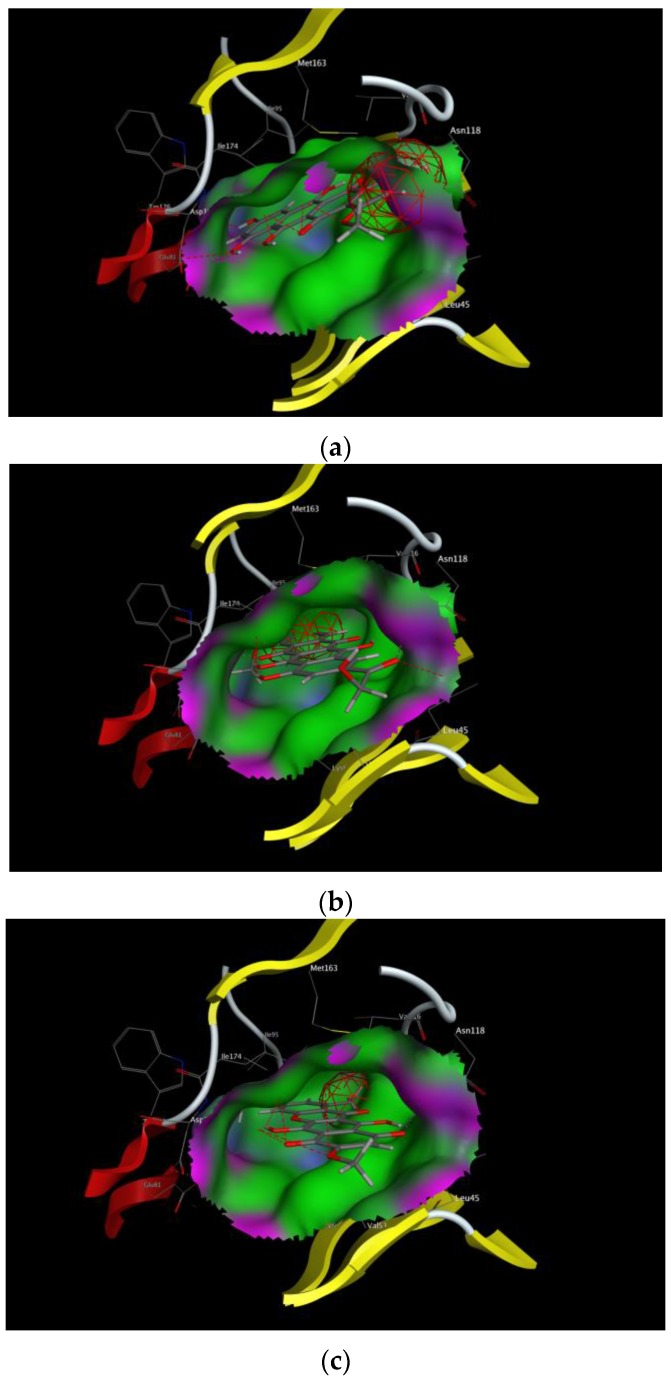
Binding mode of the selected ligands with the binding site of CK2. (**a**) ZINC35643753; (**b**) ZINC44136135; (**c**) ZINC05765165.

In order to select the best candidates among the hit compounds, docking studies of the selected compounds against the ATP active site of the CK2 kinase were performed. A conformational search for the 55 selected compounds was carried out by MOE and all the resulting 777 conformations were used for the docking study. The conformations of all structures were docked in human CK2 enzyme using the MOE. For this purpose, the 3D X-ray crystal structure of the catalytic subunit of protein kinase CK2 (PDB ID: 3C13, resolution 1.95 Å [24]) was used. All conformations were sorted according to S score (energy-based scoring method implemented in MOE) to rank the best ligand in terms of orientation and binding to the active site, the top three ranking conformations with the minimum S score were selected after visual 2D inspection. Visual 2D inspection is a common additional tool in order to exclude “false positives due to assumptions and shortcomings in docking methods and scoring function” [25], where the active site is analyzed to determine protein-ligand interaction and show exactly which atoms have tether restraints [21]. It is important to note that scoring function values might not always correlated with biological activity. Compound ZINC35643753 was ranked as a top pose followed by compound ZINC44136135 and ZINC05765165 (bikaverin) with S scores of −24.0, −23.5, and −21.1, respectively. Beside the minimum S score, each pose for each compound was analyzed to find hydrogen binding and π-π interactions. 2D interactions between receptor and ligands for the three top ranked compounds were shown in Figure 3. The three best compounds fit well in the ATP binding site as it is shown in Figure 4.

According to MOE docking, ZINC35643753 has direct bonding to Asp175, beside indirect bonding with His160, Asp120, Trp176, Glu81 and Asn161 via water molecules, Figure 3a, while ZINC44136135 shows direct interaction with Asp195 and Asn118, and arene-arene interaction with Phe113, as well as two indirect bonding with Leu45 and Asn118, via water molecules (Figure 3b). ZINC05765165 has interaction via hydrogen bonding with Asn118, as well as two indirect interactions with His160 and Asp120 via water molecules (Figure 3c). From the docking poses of the best selected compounds, it is clear that that docking strongly proposes the interaction between some functional groups of the compounds and some amino acid residues from the enzyme, and this is in line with known ATP kinase inhibitors. It might indicate that the compounds fit well in the ATP binding site of the enzyme.

**Figure 5:**
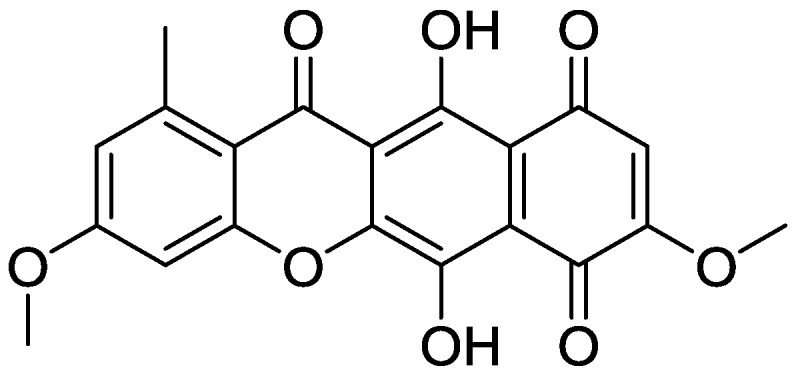
The chemical structure of bikaverin (6,11-dihydroxy-3,8-dimethoxy-1-methylbenzo[*b*]xanthene-7,10,12-trione), first isolated from the culture of *Fusarium vasinfectum* [28].

Compound ZINC35643753 and compound ZINC44136135 are commercially available compounds as all compounds included in the ZINC database, both are anthracycline analogues. This group of compounds is among the most effective drugs used in chemotherapy for treatment of both solid tumors and leukemia in adults and children. Nevertheless those compounds suffer from several severe adverse effects, especially cardiotoxicity [26]. From the three compounds identified by ZINC database mining only bikaverin (6,11-dihydroxy-3,8-dimethoxy-1-methylbenzo[*b*]xanthene -7,10,12-trione, Figure 5) was chosen for further experiments. Compound ZINC35643753 and compound ZINC44136135 belong to a group of drugs known to have severe toxicities as it is mention above, while bikaverin is a natural compound with different biological activities. The fact that bikaverin is a natural compound with antitumor activity encourage us to investigate whether this activity might be related to CK2 inhibitory activity [27].

**Figure 6:**
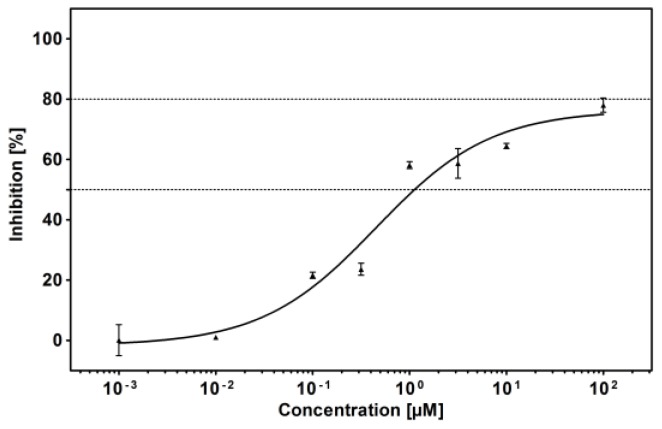
Determination of the IC_50_ value towards recombinant human CK2 of bikaverin (6,11-dihydroxy-3,8-dimethoxy-1-methylbenzo[*b*]xanthene-7,10,12-trione), the hit compound identified by ZINC database mining.CK2 holoenzyme was pre-incubated with different bikaverin concentrations (0.001–100 μM) and subsequently the in vitrophosphorylation of CK2 specificsubstrate peptide was determined by CE [30]. Relative CK2 activity at each inhibitor concentration is given in a dose-response diagram. IC_50_ values were determined in three independent replications and mean values with corresponding standard deviations are given.

Bikaverin was tested on inhibition with purified human protein kinase CK2 expressed in *E. coli* as described before [29]. For this purpose eightconcentrations rangingfrom 0.001 µM to 100 µM of bikaverin were used in a capillary electrophoresis (CE)-based activity assay [30] in comparison the enzyme without inhibitor. The obtained dose-response curve is shown in Figure 6, and was used to determine the IC_50_ value of bikaverin, which turned out to be 1.24 µM. The IC_50_ value was determined in three independent biological replicates with basically similar results (1.29, 1.30 and 1.12 µM) the mean value was determined. It is important to note that all attempts to achieve 100% inhibition were not successful. This was not due to limited solubility of the compound. As it is shown in Figure 6, maximum activity with the highest concentration of bikaverin was 80%, and the relative IC_50_ value was 0.78 µM.

## 3. Discussion

In this study a pharmacophore model for ATP-competitive inhibitors of human protein kinase CK2 was developed on the basis of known inhibitors with an indeno[1,2-*b*]indole scaffold. This model has been challenged against a set of compounds and was able to select most active compounds and excluded most nonactive ones, which indicate its validity. By using this model for database mining using the ZINC compound database, bikaverin ZINC05765165 was identified as a hit. By testing this natural compound with recombinant human CK2 it turned out to have an IC_50_ value of 1.24 µM. Bikaverin, also known as lycopersin [31], is a reddish pigment produced by different fungal species. Chemically it is a polyketide with a tetracyclic benzoxanthone structure.It has been reported to possess diverse biological activities e.g., to have antibiotic, antifungal and anticancer properties [27]. Although the antitumoral activity of bikaverin has been reported, only few reports are focusing on its mode of action and up to know, no inhibition of CK2, as a possible target, was investigated. For these reasons we chose it for in vitro inhibition determination. Our in vitro test for this compound proved that it is active and can clearly inhibit the CK2, which is an evidence of the validity for the developed pharmacophore model.

In further studies bikaverin could be used for structural modification in order to improve its inhibitory towards CK2. Further studies are necessary to test the effects of some derivatives of this compound such as acetylated derivatives or dibromo-*O*-methylbikaverin, as those compounds were more cytotoxic than bikaverin in cell lines such as EAC cells [27,32]. Also further studies to test other selected structures from the 55 compounds and modify them accordingly is planned with the hope of finding new highly active and selective inhibitor of CK2. Actually, the aim of this study was to primary in silico filter the database and try to introduce new backbones serving as possible new hits for human CK2 which was performed by discovering that bikaverin is an active CK2 inhibitor with inhibitory activity comparable to other natural inhibitors of the target enzyme such as emodin which has an IC_50_ value of 0.58 µM in our test system.

## 4. Materials and Methods

### 4.1. The Chemical Compounds

All compounds used in this study except bikaverin were described by us recently. The synthesis procedures to access to our target indeno[1,2-*b*]indoles have been published previously [9,10,12,13], bikaverin was purchased from Sigma-Aldrich (Munich, Germany).

### 4.2. In Vitro Assay

All indeno[1,2-*b*]indoles were tested for their inhibitory activity towards the human CK2 holoenzyme following the procedure described earlier [29]. The synthetic peptide RRRDDDSDDD was used as the substrate, which is reported to be most efficiently phosphorylated by CK2. The purity of the CK2 holoenzyme was superior to 99%. For initial testing, inhibition was determined relative to the controls at inhibitor concentrations of 10 μM in DMSO as a solvent. Therefore, 2 μL of the dissolved inhibitors (stock solution in DMSO) were mixed with 78 μL of CK2-supplemented kinase buffer which was composed of 1 μg CK2 holoenzyme, 50 mM Tris/HCl (pH 7.5), 100 mM NaCl, 10 mM MgCl_2_ and 1 mM DTT. The reaction was started by the addition of 120 μL assay buffer, which was composed of 25 mM Tris/HCl (pH 8.5), 150 mM NaCl, 5 mM MgCl_2_, 1 mM DTT, 100 μM ATP and 0.19 mM of the substrate peptide RRRDDDSDDD. The reaction was carried out for 15 minutes at 37 °C and stopped by the addition of 4 μL EDTA (0.5 M). Subsequently the reaction mixture was analyzed by a PA800 capillary electrophoresis from Beckman Coulter (Krefeld, Germany). Acetic acid (2 M, adjusted with conc. HCl to a pH of 2.0) was used as the electrolyte for electrophoretic separation. The separated substrate and product peptide were detected at 214 nm using a DAD-detector. Pure solvent was used as negative control (0% inhibition), assays without the enzyme were used as positive control (100% inhibition). Compounds with at least 50% inhibition at 10 μM were used for IC_50_ determinations. For the determination of IC_50_, inhibition was determined using nine inhibitor concentrations ranging from 0.001 μM to 100 μM. IC_50_ were calculated from the resulting dose-response curves [30].

### 4.3. Computational Methods

#### 4.3.1. Pharmacophore Generation

Molecular Operating Environment software package (MOE, Chemical Computing Group, Montreal, QC, Canada) was used to perform this study [21] running on an Intel Core, Duo based, 2.26 GHz processor.The molecular structures of the inhibitors were built with MOE and energy was minimized in the MMFF94x force field as implemented in the software. In order to develop a reliable pharmacophore model to identify new CK2 inhibitors, a database of known indeno[1,2-*b*]indole inhibitors was created. The structures of the inhibitors together with their inhibitory activity were published by us as mentioned above. In total this data base contained 50 compounds (Table 1, Table 2 and Table 3). The pharmacophore model was developed by aligning the minimized structures of the five most active compounds in the database namely compounds **1**–**5** with IC_50_ values of 25, 170, 360, 430, and 610 nM, respectively (training set). This alignment was obtained using MOE’s flexible alignment and all conformations of the molecules were considered for the alignment [21]. Due to the relatively identical backbones of the compounds, the selected compounds were very well aligned (Figure 1). A conformational search was performed for each of the 50 compounds using the MMFF94x force field within the MOE to generate a multi-conformer database (test set). The search was performed using the default settings of MOE, and a query was developed based on the alignment of the five most active compounds (training set). The Consensus query of MOE was used to create all necessary features of the hypothetical pharmacophore model, tolerance was set at 1 and threshold was set at 80%. In order to optimize this multi features model, each individual pharmacophore feature was removed and the test set was scanned using the remaining features. Nine features were necessary as mentioned earlier (Figure 2), one Pi ring center on the B ring, one hydrophobic centroid, two aromatic center/hydrophobic atoms on ring A and C, one Pi ring center/aromatic/hydrophobic centroid on ring D, and two hydrogen bond acceptors on rings B and D, together with two hydrogen acceptor projections, beside one excluded volume. The radius of F1 and F3 were set at 0.8 A°, F2 was set at 0.6 A°, F4, F9 were set on 1 A°, F5 was set on 0.4 A°, F6 was set on 0.5 A°, F7 and F8 were set on 0.7 A°, and V1 was set on 1 A° (Figure 2).The pharmacophore search function of MOE was used to scan the test set using all default options of MOE.

#### 4.3.2. Database Search

ZINC Database is a free database of commercially available compounds for virtual screening. It contains over 35 million purchasable compounds in ready-to-dock, 3D formats. ZINC is provided by Department of Pharmaceutical Chemistry at the University of California, San Francisco [20]. The compounds in the ZINC Database are organized in several categories namely, Standard: for delivery within 10 weeks, including in-stock and make on demand compounds; Clean: where stricter filtering rules have been applied, in stock: for immediate delivery; and Boutique: expensive selection of compounds not included in the former categories, and each of these categories is also redivided into Lead-like, Fragment-like, Drug-like and All. Since we are trying to find an active structure which can be directly purchasable, we chose in this work the “All Now” (all purchasable in 2 weeks) database which contains around 13 million compounds and was used to search for compounds that fit to most features of the developed pharmacophore. The compounds from the ZINC database were downloaded into MOE then used for the screening. The selected list of compounds from the database was docked against CK2 enzyme using the MOE software package.

#### 4.3.3. Receptor Refinement

Three dimensional 3D structure of the CK2 complex with emodin was obtained from the Protein Data Bank (PDB) using PDB ID: (3C13) having a resolution of 1.95 Å. The structure was optimized by adding hydrogen atoms using the MOE software [21]. Then water molecules were removed from the structure and 3D protonation was done to change the state into ionization level. In the second step, energy minimization was performed using defaults parameters, where the force field was MMFF94x.

#### 4.3.4. Database Generation

The selected compounds from the database were rebuilt with MOE building option implemented in the software. The compounds were optimized by adding hydrogen atoms using the option of MOE software. Energy of the compounds was minimized using the following parameters gradient: 0.05, Force Field: MMFF94X, Chiral constraint and Current Geometry. The conformation methodology was used to develop low energy conformations for each compound, applying the LowModMD method with RMS gradient of 0.05, all other parameters were used as default. All of the compounds and their conformations were saved in mdb database and later employed for docking studies.

#### 4.3.5. Molecular Docking

The docking of the selected compounds from the database (compounds **1**–**55**) into the active site of CK2 enzyme (3C13) was achieved using MOE-Dock implemented on MOE. The docking parameters were set as Rescoring 1: London dG, Placement: triangle matcher, Retain 30, Refinement Force field, and Rescoring 2: London dG. Docking part of MOE can give correct conformation of the ligand to obtain minimum energy structure. The top conformation for each compound was selected based on the S score and visual inspection was carried out by Lgplot implemented in MOE. Compounds showing significant interaction with the residues of binding pocket of CK2 were picked as promising hits. Prior to dock, the initial ligand from the complex structure was extracted.For the scoring function, lower scores indicate more favorable poses. The unit for the scoring function is Kal/mol, and the S score refers to the final score, which is the score of the last stage that was not set to None. The Lig X function in MOE was used for conducting interactive ligand modification and energy minimization in the active site of the receptor.

## 5. Conclusions

In this study we were able to valorize the library of indeno[1,2-*b*]indoles designed for CK2 inhibition to determine the necessary ligand binding requirement for ATP binding CK2 inhibitors. We were also able to use the new model for searching in ZINC database. We discovered a potential inhibitor of CK2 and we experimentally confirmed its potency to inhibit CK2. Bikaverin can be regarded as a hit scaffold for designing CK2 inhibitors. It is also possible to use the pharmacophore model to further identify new inhibitors for the target enzyme from other databases, and modify the selected structures accordingly. Finally discovering that bikaverin is a CK2 inhibitor is an important finding and provides an important contribution toward finding active and selective CK2 inhibitors.

## Figures and Tables

**Table 1:**
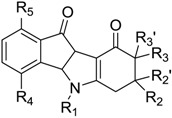
Structures of the ketonic indeno[1,2-*b*]indoles and their IC_50_ values towards CK2, which were used for the training and test sets, data from Alchab et al. [9] and Gozzi et al. [13].

Code	R_1_	R_2_	R_2′_	R_3_	R_3′_	R_4_	R_5_	IC_50_ (µM)
**1**	CH(CH_3_)_2_	H	H	H	H	O–CH_2_CH=C(CH_3_)_2_	H	0.025
**2**	CH(CH_3_)_2_	CH_3_	H	H	H	H	H	0.17
**3**	CH(CH_3_)_2_	H	H	H	H	H	H	0.36
**5**	CH(CH_3_)_2_	CH(CH_3_)_2_	H	H	H	H	H	0.61
**8**	CH_2_CH_2_(*ortho*-OMe)Ph	H	H	H	H	H	H	1.40
**9**		H	H	H	H	H	H	1.44
**14**	CH_2_CH_2_C_6_H_5_	CH_3_	H	H	H	H	H	2.50
**18**	CH_2_CH_2_(*para*-OMe)Ph	H	H	H	H	H	H	4.10
**22**	CH_2_CH_2_(*meta*-OMe)Ph	H	H	H	H	H	H	5.10
**23**	CH_2_CH_2_ CH_2_C_6_H_5_	H	H	H	H	H	H	6.00
**24**	CH_2_CH_2_C_6_H_5_	H	H	H	H	H	H	7.00
**26**	CH(CH_3_)_2_	H	H	CH_3_	H	H	H	9.20
**28**	CH(CH_3_)_2_	H	H	(CH_2_Ph)_2_	CH_2_Ph	H	H	>10
**29**	CH_2_(*para*-OMe)Ph	H	H	H	H	H	H	>10
**32**	CH(CH_3_)_2_	H	H	H	H	H	O–CH_2_CH=C(CH_3_)_2_	>10
**37**	CH(CH_3_)_2_	(*para*-F)Ph	H	H	H	H	H	>10
**39**	CH(CH_3_)_2_	furan-2-yl	H	H	H	H	H	>10
**40**	CH_2_Ph	H	H	H	H	H	H	>10
**41**	CH_2_CH_2_Ph	Ph	H	H	H	H	H	>10
**43**	H	CH_3_	H	COOCH_3_	H	H	H	>10
**44**	H	H	H	H	H	H	H	>10
**45**	CH_2_Ph	CH_3_	H	COOCH_3_	H	H	H	>10
**46**	CH(CH_3_)_2_	H	H	CH(CH_3_)_2_	H	H	H	>10
**47**	CH_2_Ph	CH_3_	CH_3_	H	H	H	H	>10
**48**	CH_2_Ph	CH_3_	H	H	H	H	H	>10
**49**	CH(CH_3_)_2_	Ph	H	H	H	H	H	>10
**50**	CH_2_Ph	Ph	H	H	H	H	H	>10

**Table 2:**
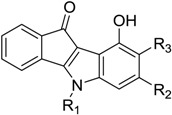
Structures of the phenolic indeno[1,2-*b*]indoles and their IC_50_ values towards CK2, which were used for the training and test sets, data from Hundsdorfer et al. [10], and Gozzi et al. [12].

Code	R_1_	R_2_	R_3_	IC_50_ (µM)
**6**	CH(CH_3_)_2_	CH_3_	H	1.27
**7**	CH_2_CH_2_(*ortho*-OMe)Ph	H	H	1.30
**10**	CH(CH_3_)_2_	CH(CH_3_)_2_	H	1.65
**12**	CH(CH_3_)_2_	H	H	2.00
**15**	CH(CH_3_)_2_	(*para*-F)Ph	H	2.77
**16**	CH(CH_3_)_2_	furan-2-yl	H	3.63
**25**	CH_2_CH_2_C_6_H_5_	H	H	7.50
**27**	CH_2_Ph	Ph	H	>10
**30**	CH_2_CH_2_C_6_H_5_	CH_3_	H	>10
**33**	CH(CH_3_)_2_	H	CH_3_	>10
**38**	CH(CH_3_)_2_	H	CH(CH_3_)_2_	>10
**42**	CH_2_Ph	H	H	>10

**Table 3:**
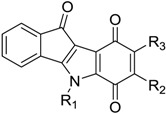
Structures of the quinonic indeno[1,2-*b*]indoles and their IC_50_ values towards CK2, which were used for the training and test sets, data from Alchab et al. [9], Gozzi et al. [12].

Code	R_1_	R_2_	R_3_	IC_50_ (µM)
**4**	CH(CH_3_)_2_	CH_3_	H	0.43
**11**	CH(CH_3_)_2_	furan-2-yl	H	1.65
**13**	CH_2_C_6_H_5_	H	H	2.20
**17**	CH_2_CH_2_C_6_H_5_	CH_3,_	H	4.10
**19**	CH(CH_3_)_2_	CH(CH_3_)_2_	H	4.76
**20**	CH(CH_3_)_2_	H	CH_3_	4.90
**21**	CH(CH_3_)_2_	H	H	5.50
**31**	CH(CH_3_)_2_	H	CH(CH_3_)_2_	>10
**34**	CH(CH_3_)_2_	(*para*-F)Ph	H	>10
**35**	CH(CH_3_)_2_	Ph	H	>10
**36**	CH_2_CH_2_Ph	H	H	>10

## Supplementary Materials

The following is available online at http://www.mdpi.com/1424-8247/10/1/8/s1, Table S1.
