# Supplementary material for: Development of Pharmacophore Model for Indeno[1,2-b]indoles as Human Protein Kinase CK2 Inhibitors and Database Mining
Source: Pharmaceuticals (Basel). 2017 Jan 9;10(1):8. doi: 10.3390/ph10010008 (PMC5374412; doi:10.3390/ph10010008)
Supplement: Supplementary file 1 [file pharmaceuticals-10-00008-s001.pdf]

pharmaceuticals

Supplementary Materials: Development of Pharmacophore Model for Indeno[1,2-*b*] indoles as Human Protein Kinase CK2 Inhibitors and Database Mining

Samer Haidar, Zouhair Bouaziz, Christelle Marminon, Tuomo Laitinen, Antti Poso, Marc Le Borgne and Joachim Jose

Table S1. Chemical structures of the (hit) compounds from 3D mining of ZINC database using the pharmacophore features of the indeno[1,2-*b*] indole CK2 inhibitors together with the corresponding S scores.

Code	Compound Structure	S Score	Code	Compound Structure	S Score
**01893208**	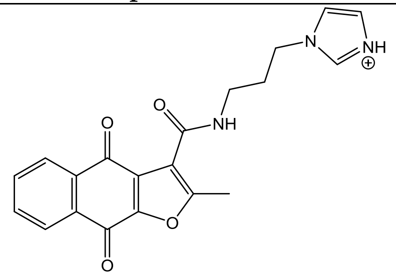	**−14.9665**	**44136135**	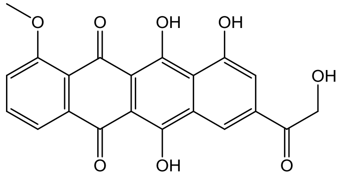	**−23.6586**
**37867960**	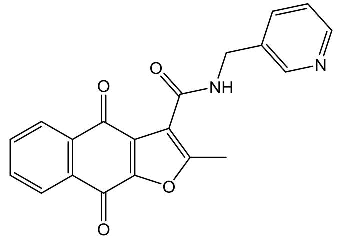	**−13.8396**	**01576029**	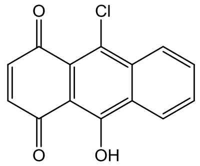	**−15.7471**
**02700659**	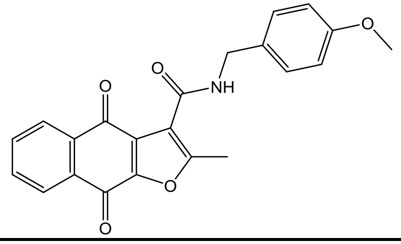	**−13.9335**	**05765165**	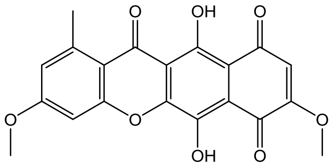	**−21.3049**

**05386044**	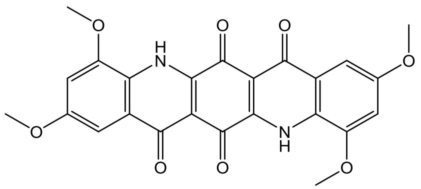	**−20.8452**	**01608278**	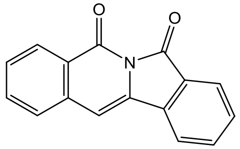	**−13.7865**
**06576067**	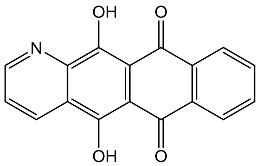	**−16.6434**	**02941398**	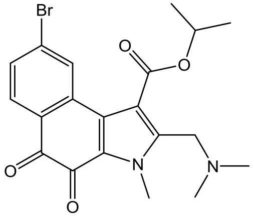	**−14.3634**
**01214105**	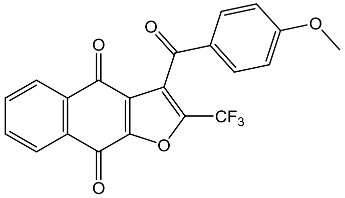	**−14.0689**	**05002851**	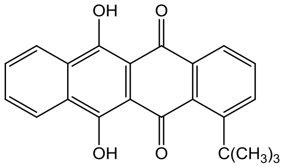	**−18.6287**
**00082235**	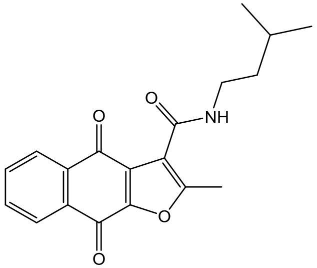	**−13.1032**	**04981633**	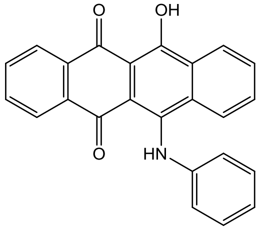	**−17.0522**
**05604841**	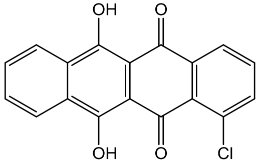	**−17.6656**	**0079314**	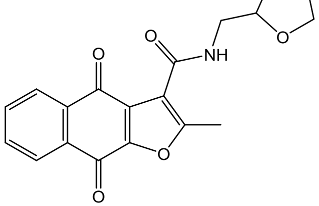	**−14.7410**

**01236034**	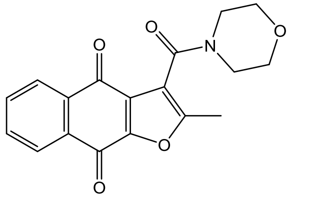	**−14.5727**	**13378773**	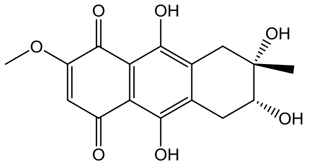	**−18.4495**
**01236036**	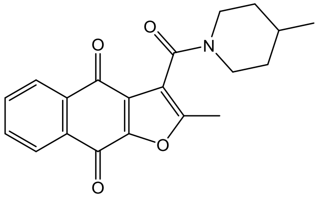	**−12.6232**	**01613555**	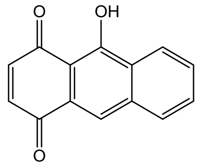	**−16.4633**
**01236042**	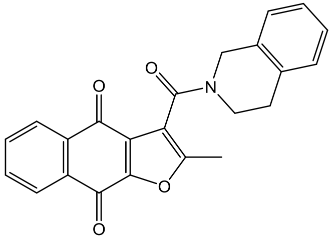	**−12.7093**	**00720964**	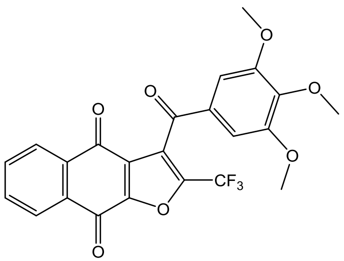	**−17.5191**
**01236043**	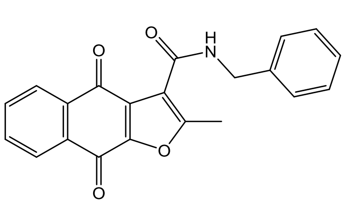	**−14.5567**	**73816406**	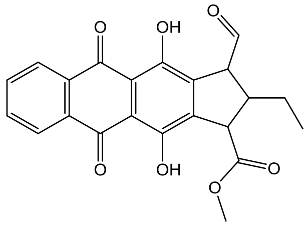	**−19.1375**
**01236046**	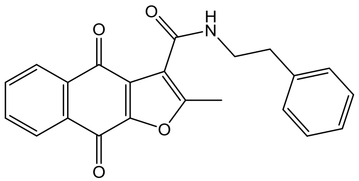	**−13.9202**	**73816415**	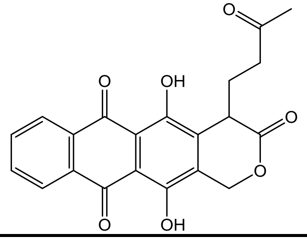	**−19.8413**

**02700667**	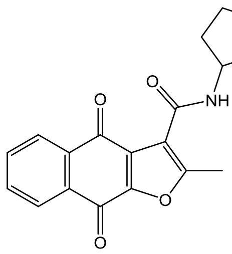	**−14.2688**	**73816487**	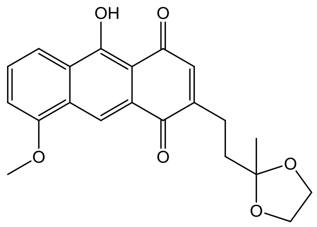	**−18.3351**
**00978111**	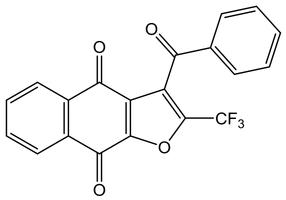	**−13.7927**	**73816498**	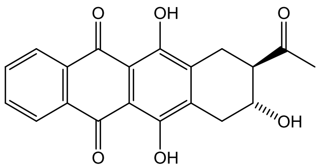	**−20.1982**
**53166374**	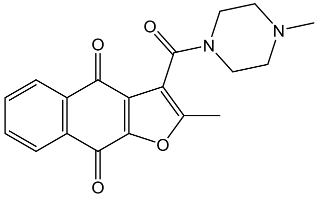	**−12.7809**	**73816500**	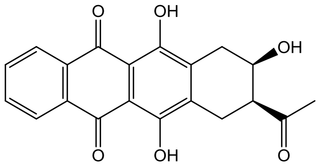	**−18.2989**
**00065262**	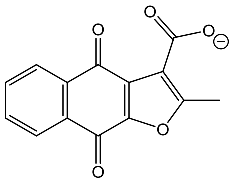	**−14.2690**	**73816512**	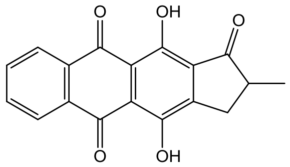	**−17.7249**
**35643753**	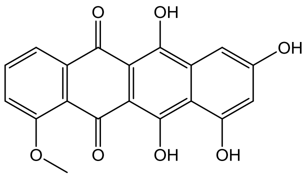	**−24.0183**	**02074909**	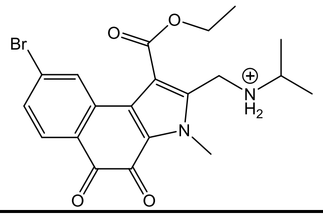	**−15.3759**

**04776566**	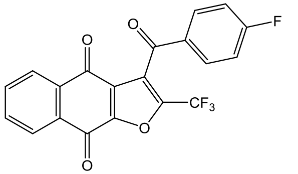	**−14.2474**	**44547628**	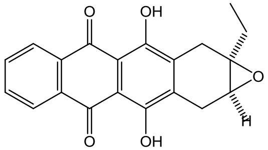	**−20.2288**
**04026692**	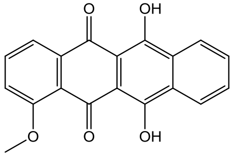	**−18.3439**	**44547636**	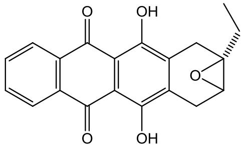	**−19.1737**
**00449156**	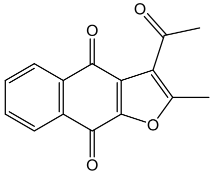	**−11.8459**	**03051841**	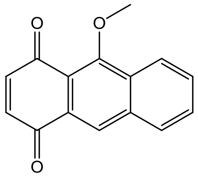	**−12.8899**
**05425576**	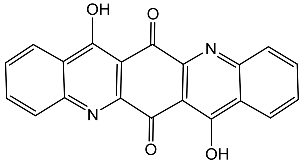	**−19.0233**	**05086137**	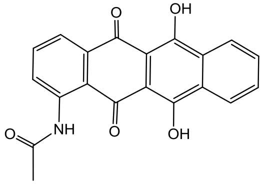	**−17.6893**
**10034949**	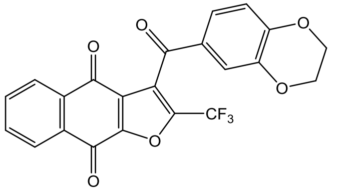	**−16.1686**	**0079314**	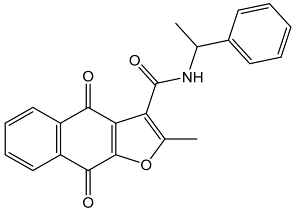	**−14.7410**
**10034950**	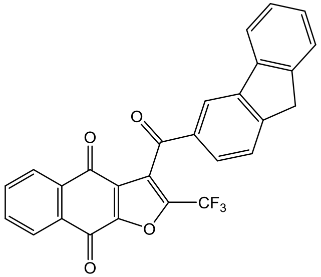	**−14.1934**	**02256986**	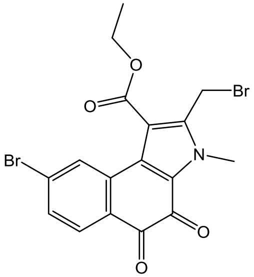	**−13.8326**

**39124410**	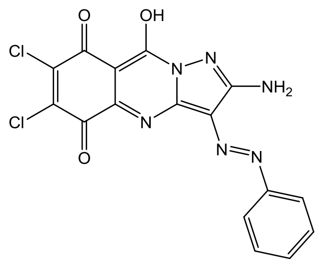	**−18.6799**	**16952888**	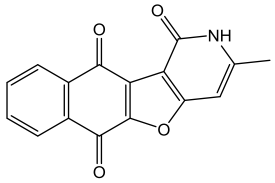	**−12.6946**
**10034955**	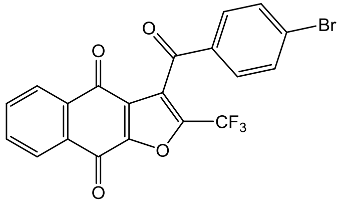	**−13.8656**	**00721112**	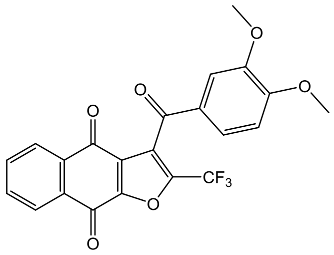	**−14.9330**
**01214623**	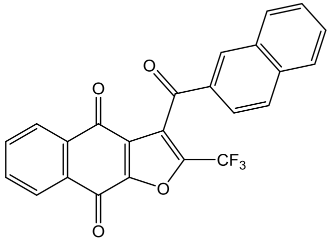	**−14.2635**	**85409391**	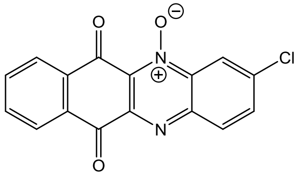	**−16.5118**
			**05776381**	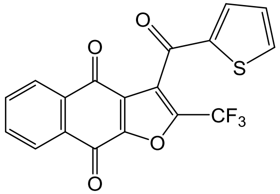	**−14.1791**
